# Smad7 as a positive regulator of intestinal inflammatory diseases

**DOI:** 10.1016/j.crimmu.2023.100055

**Published:** 2023-01-24

**Authors:** Giovanni Monteleone, Federica Laudisi, Carmine Stolfi

**Affiliations:** Department of Systems Medicine, University of Rome “Tor Vergata”, Director of Gastroenterology Unit, Department of Medical Science, Policlinico Universitario Tor Vergata, Rome, Italy

**Keywords:** Crohn’s disease, Ulcerative colitis, Inflammatory bowel diseases, TGF-beta

## Abstract

In physiological conditions, the human gut contains more immune cells than the rest of the body, but no overt tissue damage occurs, because several regulatory mechanisms control the activity of such cells thus preventing excessive and detrimental responses. One such mechanism relies on the action of transforming growth factor (TGF)-β1, a cytokine that targets both epithelial cells and many immune cell types. Loss of TGF-β1 function leads to intestinal pathology in both mice and humans. For instance, disruption of TGF-β1 signaling characterizes the destructive immune-inflammatory response in patients with Crohn’s disease and patients with ulcerative colitis, the major human inflammatory bowel disease (IBD) entities. In these pathologies, the defective TGF-β1-mediated anti-inflammatory response is associated with elevated intestinal levels of Smad7, an antagonist of TGF-β1 signaling. Consistently, knockdown of Smad7 restores TGF-β1 function thereby attenuating intestinal inflammation in patients with IBD as well as in mice with IBD-like colitis. Up-regulation of Smad7 and reduced TGF-β1 signaling occurs also in necrotizing enterocolitis, environmental enteropathy, refractory celiac disease, and cytomegalovirus-induced colitis. In this article, we review the available data supporting the pathogenic role of Smad7 in the gastrointestinal tract and discuss whether and how targeting Smad7 can help attenuate detrimental immuno-inflammatory responses in the gut.

## Highlights

• Defective TGF-β-driven immunosuppression results in chronic intestinal inflammation.
• Smad7, a TGF-β signaling inhibitor, is locally but not systemically up-regulated in many intestinal inflammatory disorders.
• Knockdown of Smad7 restores TGF-β signaling and attenuates gut inflammation.

## Graphical abstract

**Figure:**
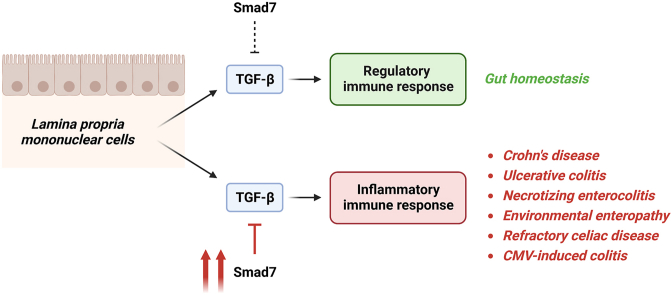

## 1 Introduction

The gastrointestinal tract harbors a large number of commensal microorganisms (i.e. bacterial, viral, and fungal species) that induce maturation of the mucosal immune system. Indeed, the gastrointestinal mucosa contains more immune cells than the rest of the body, and this state of “physiological inflammation”, together with the barrier effect of the epithelium, contributes to providing resistance to invading pathogens, without altering the normal absorptive and digestive functions (MacDonald and Monteleone, 2005). Studies in murine models of intestinal inflammation and patients with chronic colitis indicate that several regulatory mechanisms control the epithelial integrity and activity of the immune cells thereby maintaining gut homeostasis (MacDonald et al., 2011). Consistently, defects in the epithelial barrier and/or lack of function of regulatory molecules can break the immune tolerance toward the luminal microflora thus resulting in chronic gut inflammation (MacDonald and Monteleone, 2005). This occurs, for instance, in mice lacking the regulatory cytokine interleukin (IL)-10 (Glocker et al., 2009) as well as in humans with genetic deficiencies in IL-10 and IL-10 receptors (Asseman et al., 1999). Similarly, mice whose T cells do not respond to transforming growth factor (TGF)-β1, another regulatory cytokine, and mice null for TGF-β1 succumb to severe and widespread autoimmunity, involving also the intestines (Gorelik and Flavell, 2000). In the mammalian gut, epithelial cells and several immune cells, particularly dendritic cells (DC), produce TGF-β1. The cytokine is secreted in an inactive form bound to the latency-associated peptide (LAP) and activation of TGF-β1 needs conformational changes or protein cleavage of LAP, a phenomenon mediated by integrins αvβ6 and αvβ8, furin, and metalloproteinases (Bauche and Marie, 2017). The TGF-β1 function is initiated by its binding to a transmembrane receptor with serine/threonine kinase activity, named TGF-β1 Type 2 receptor (TβR2). Interaction between TGF-β1 and TβR2 causes the auto-phosphorylation of the receptor and subsequent recruitment and phosphorylation of TβR1. Although TGF-β1 can activate various intracellular pathways (e.g. MAP kinases and AKT/PI3K) most of its regulatory effects rely on the ability of the activated TβR1-TβR2 complex to phosphorylate Smad2 and Smad3. Following their phosphorylation, these two proteins interact with Smad4, leading to the formation of a Smad2/Smad3-Smad4 complex that moves to the nucleus to regulate the transcription of several genes (Massague et al., 2005; Piek et al., 1999). Circumstantial evidence indicates that, in the normal human gastrointestinal tract, TGF-β1 works properly. Indeed, lamina propria mononuclear cells (LPMC) isolated from the normal, uninflamed colon of individuals undergoing colonoscopy for colorectal cancer screening programs, express constitutively phosphorylated Smad3. Moreover, incubation of normal intestinal mucosal explants with a neutralizing TGF-β1 antibody increases the expression of inflammatory cytokines (e.g. interferon-γ) and transcription factors (i.e. T-bet), which are known to be negatively regulated by TGF-β1 in T cells (Di Sabatino et al., 2008). Finally, treatment of normal LPMC with recombinant TGF-β1 inhibits NF-kB activity, thereby suppressing inflammatory cytokine synthesis (Monteleone et al., 2004).

**Fig. 1:**
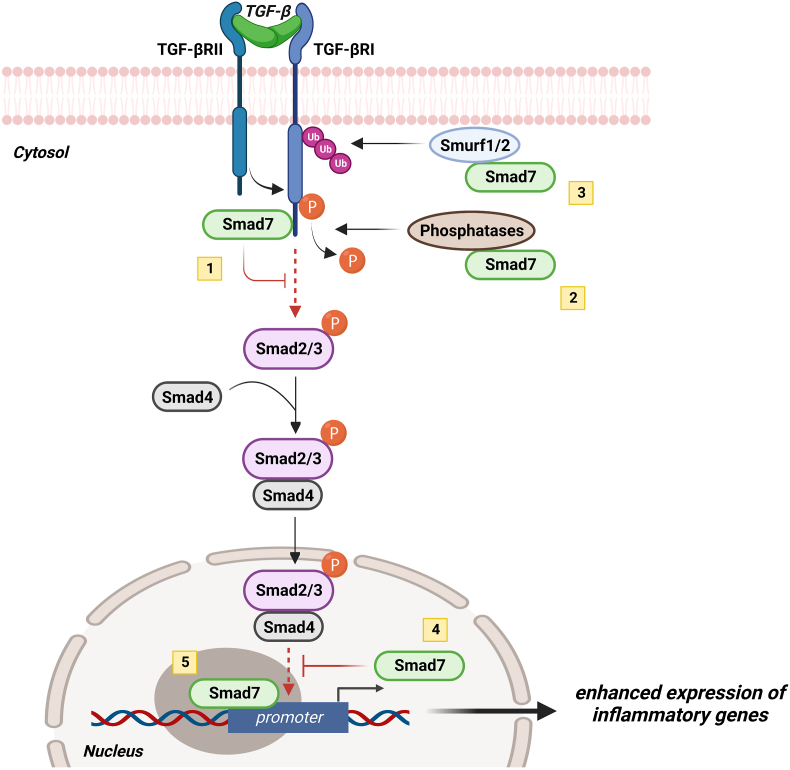
Smad7 enhances the expression of inflammatory genes by both negatively regulating TGF-β1 signaling and controlling DNA promoter activity. These Smad7-driven effects are mediated through various mechanisms: 1. By binding to TβR1 prevents Smad2/3 phosphorylation; 2. De-phosphorylation and inactivation of TβR1 through recruitment of phosphatases; 3. Interaction with E3 ubiquitin ligases SMURF1/2 to pro-mote proteasome-mediated degradation of TβR1; 4. In the nucleus, inhibition of the association of Smad2-3/Smad4 complex with specific Smad-responsive DNA sequences; 5. In the nucleus, by binding DNA promoters. Created with Biorender.com.

Another Smad protein, termed Smad7, regulates negatively TGF-β1-associated Smad signaling through various mechanisms (Fig. 1). For instance, Smad7 can bind to TβR1 and prevent Smad2/3 phosphorylation (Hayashi et al., 1997; Nakao et al., 1997). Smad7 can promote the recruitment of phosphatases to TβR1 and favor de-phosphorylation and inactivation of the receptor (Shi et al., 2004) as well as proteasome-mediated degradation of TβR1 in cooperation with SMURF1/2, two E3 ubiquitin ligases (Ebisawa et al., 2001; Kavsak et al., 2000). Smad7 can also localize into the nucleus and limit the association of Smad2-3/Smad4 complex with specific Smad-responsive DNA sequences (Zhang et al., 2007). More recent studies have shown that Smad7 can exert some regulatory effects on the expression and function of molecules involved in the regulation of inflammatory response (e.g. Stat3 expression/activation) via a TGF-β1-independent mechanism (Maresca et al., 2022).

In this article, we review the available data supporting the pathogenic role of Smad7 in the gastrointestinal tract and discuss whether and how targeting Smad7 can help attenuate detrimental immuno-inflammatory responses in the gut.

## 2 Expression of Smad7 in IBD

Ulcerative colitis (UC) and Crohn’s disease (CD) are chronic, relapsing IBDs (Abraham and Cho, 2009). CD usually involves the terminal ileum and colon, but it can present anywhere in the alimentary tract, from the mouth to the anus, with lesions transmural and discontinuous and histologically characterized by the accumulation of inflammatory cells, and by deep-fissuring ulcers. Fibrosis in the deeper layers of the affected intestinal wall contributes to the development of strictures, as a result of the overproduction of collagen by fibroblasts and smooth muscle cells (Xavier and Podolsky, 2007). In contrast, in UC, the pathological process involves the mucosa and, in some circumstances, the submucosal layer, and the inflammatory lesions arise in the rectum and can extend proximally in a continuous fashion resulting in variable degrees of involvement of the colon. UC-related inflammation is marked by the accumulation of neutrophils, lymphocytes, plasma cells, and macrophages in the lamina propria. Neutrophils can invade the epithelium and cross into the crypts, giving rise to cryptitis and crypt abscesses (Xavier and Podolsky, 2007). Both CD and UC can be complicated by extra-intestinal manifestations (Marotto et al., 2020).

The etiology of UC and CD is not yet known and therefore no curative therapy exists. The most accredited pathogenic hypothesis is that IBDs are triggered by multiple environmental agents that, in genetically susceptible individuals, promote an excessive immune response against luminal antigens, which is poorly controlled by counter-regulatory mechanisms (Marafini et al., 2019; Pallone and Monteleone, 2001).

In this context, our previous studies showed that in IBD there is a defective TGF-β1 activity. Indeed, IBD LPMCs contain reduced levels of phosphorylated Smad3 as compared to normal LPMCs and are unresponsive to the suppressive action of recombinant TGF-β1 in terms of cytokine production (Monteleone et al., 2001, 2004). A further line of evidence is provided by the demonstration that CD4^+^ T cells isolated from the inflamed gut of CD patients are resistant to regulatory T cell-mediated suppression, a phenomenon that relies on intact TGF-β1 signaling. These findings have been linked to the elevated expression of Smad7 in the inflamed intestinal mucosa of IBD patients (Monteleone et al., 2001). Time-course studies performed in CD patients with postoperative recurrence showed also that mucosal induction of Smad7 precedes the appearance of endoscopic lesions, suggesting a role for the protein in the very early events leading to the mucosal damage (Zorzi et al., 2020). In IBD, over-expression of Smad7 occurs at the protein but not RNA level, indicating post-transcriptional regulation of the molecule (Monteleone et al., 2005). Indeed, there is evidence that, in IBD, Smad7 undergoes acetylation, a dynamic post-translational modification that prevents proteasome-mediated ubiquitination-driven degradation (Monteleone et al., 2005). The predominant IBD-associated Smad7 acetylation relies on the enhanced activity of the transcriptional coactivator p300 and reduced expression of SIRT1, a member of the mammalian Sirtuin family, which normally deacetylates the lysine residues of Smad7 (Caruso et al., 2014a).

## 3 Smad7 contributes to sustaining pathogenic signals in the gut

In the last two decades, many studies have shed light on the pathogenic role of Smad7 in the gut. By using a specific antisense oligonucleotide that hybridizes to Smad7 mRNA in a sequence-specific manner, thereby triggering RNase H1 activity, mRNA degradation, and protein inhibition, we were able to show that Smad7 knockdown in ex vivo organ cultures of IBD mucosal explants and LPMCs restored the TGF-β1-associated Smad3 signaling and suppressed the production of inflammatory cytokines (Monteleone et al., 2001). Additionally, Smad7 knockdown in CD4^+^ T cells isolated from the inflamed gut of CD patients made such cells responsive to the suppressive action of Tregs (Monteleone et al., 2001). Immunohistochemical analysis of IBD sections showed that Smad7 is up-regulated not only in LPMC but also in epithelial cells (Sedda et al., 2018). The exact contribution of Smad7 in the epithelial cell behavior remains to be determined, even though there is evidence that, in such cells, Smad7 co-localizes with CCL20, a chemokine involved in the recruitment of immune cells to the intestine and over-expressed in CD epithelium (Marafini et al., 2017), and Smad7 knockdown in CD mucosal explants reduces CCL20 production (Marafini et al., 2017).

In the gut, the epithelial layer acts as a physical barrier protecting the host from the luminal content (Peterson and Artis, 2014). TGF-β1/Smad signaling has a central role in both epithelial homeostasis and wound repair, and enhances epithelial barrier protection (Troncone et al., 2018). Consistently, mice lacking intestinal expression of Smad4 have increased intestinal permeability (Marincola Smith et al., 2021). Indirect evidence indicates that Smad7-induced TGF-β1/Smad signaling impairment reduces the expression of intestinal claudin-4 thereby contributing to the impaired epithelial function seen in IBD patients (Marincola Smith et al., 2021).

In line with the human data is the demonstration that Smad7 is over-expressed in the inflamed colon of mice with oxazolone-induced colitis and mice with trinitrobenzene sulfonic acid (TNBS)-induced colitis, two models showing some immunological similarities with UC and CD, respectively (Boirivant et al., 1998, 2006; Neurath et al., 1995). In both these models, Smad7 up-regulation is associated with decreased Smad3 phosphorylation, and oral administration of Smad7 AS restores TGF-β1/Smad3 signaling with the downstream effect of reducing inflammatory cytokine production and ameliorating the ongoing mucosal inflammation (Boirivant et al., 2006).

During the acute phases of human IBD and experimental colitis in mice, Smad7 is over-expressed by mucosal T cells. To further explore the role of T cell-associated Smad7 on ongoing colitis, we generated a transgenic (Tg) mouse over-expressing Smad7 selectively in T cells and NKT cells (Rizzo et al., 2011). Mice are viable and exhibit no overt gut inflammation. However, following administration of dextran sodium sulfate (DSS), which breaks the intestinal epithelial barrier thus allowing massive penetration of luminal antigens into the lamina propria, Smad7-Tg mice develop severe colitis compared to controls (Rizzo et al., 2011). Moreover, by using the T cell-transfer model of colitis, we showed that the adoptive transfer of Smad7 Tg naïve CD4^+^ T cells, in the absence of Tregs, into naïve mice induces colitis that is more severe than that documented in mice reconstituted with wild-type T cells (Fantini et al., 2009). Further analysis revealed that Smad7 Tg T cells have reduced expression of aryl hydrocarbon receptor (AhR), a transcription factor that is positively regulated by TGF-β1 and stimulates IL-22 production and consequent regulatory responses in the gut (Monteleone et al., 2011). Consistently, in the T-cell transfer colitis model, treatment of colitic mice with AhR activators attenuates the mucosal inflammation induced by wild-type T cells but does not influence colitis induced by Smad7 Tg T cells (Monteleone et al., 2016).

Tregs can be induced peripherally and intestinal CD103+ DCs are known to enhance such a differentiation (Jaensson et al., 2008; Ruane and Lavelle, 2011), a phenomenon that is mediated by the interaction between CD4^+^ T cell-associated programmed death 1 (PD1) and its ligands, PDL1, and PDL2, which are expressed on DCs. One major mechanism by which PD1 induces tolerance is by promoting Foxp3+ Tregs when engaged with PDL1-expressing APCs (Francisco et al., 2009; Liu et al., 2013). To study the effect of Smad7 on DCs, Garo and colleagues generated mice deficient in Smad7 in CD11c + DCs. Mesenteric lymph node DCs from Smad7-deficient mice present with a regulatory phenotype, expressing much higher levels of PDL1, PDL2, and CD103 (Garo et al., 2019). Such a regulatory phenotype was also seen in wild-type DCs stimulated with TGF-β1, as a result of a direct Smad3-mediated activating effect on PDL1, PDL2, and CD103 promoters. Consistently, Smad7-deficient DCs promoted the differentiation of naive CD4^+^ T cells into Foxp3+ Tregs, and mice with Smad7-deficient DCs developed less severe DSS-induced colitis compared to controls. Specifically, mice with Smad7-deficient DCs exhibited decreased inflammatory infiltrates and reduced levels of proinflammatory cytokines, and increased PDL2 and CD103 expression (Garo et al., 2019). Moreover, treatment of those mice with blocking antibodies to either PD1 or CD25 abrogated colitis resistance observed in mice with Smad7-deficient DCs, supporting the contribution of Tregs and Treg-promoting PD1 signaling in mitigating colitis severity in mice carrying on Smad7-deficient DCs. Finally, intraperitoneal administration of Smad7 to mice attenuated DSS-driven colitis and increased TGF-β1 and PDL2/1-PD1 signaling (Garo et al., 2019). The Smad7 regulation of PD1/PDL1-PDL2 signaling relies also on the ability of Smad7 to suppress TGF-β1-driven PD1 expression on T cells. Indeed, Smad7-deficient naive CD4^+^ T cells showed enhanced PD1 induction and improved Treg differentiation, in response to TGF-β and PDL1/2 stimulation, and were less pathogenic in the T-cell transfer model as compared to cells isolated from wild-type mice (Garo et al., 2019). Altogether these findings indicate that Smad7 limits PDL2/1-PD1 signaling, a major immunoregulatory axis in the intestinal Treg differentiation and immune tolerance, thus explaining one of the mechanisms by which high Smad7 sustains gut inflammation.

The natural history of CD can be complicated by fibrostrictures, which are most common in the small bowel and particularly in the terminal ileum. The pathogenesis of fibrostrictures is not fully understood but they may be due to either incomplete control of mucosal inflammation or inflammation-independent mechanisms of intestinal damage. Studies in experimental models of intestinal fibrosis and descriptive evaluation of molecular signatures present in the CD strictures suggest that TGF-β1/Smad signaling regulates multiple steps of the fibrogenic process (Stolfi et al., 2020). By using a mouse model of established colitis-driven intestinal fibrosis, we have recently shown that oral administration of Smad7 AS to colitic mice attenuated colitis thus limiting the degree of intestinal fibrosis (Izzo et al., 2018). However, a somehow different scenario emerges from studies by Schuler and colleagues, who used mice with a systemic knockout of Smad7 in all the cells. The authors showed that the absence of Smad7 attenuated colitis but increased fibrosis (Schuler et al., 2022). The reason for this discrepancy remains unknown but differences in the genetic backgrounds of the animals used in the studies mentioned and/or in the experimental settings could have influenced the outcomes of fibrogenesis.

## 4 High Smad7 marks inflammation in other intestinal disorders

Necrotizing enterocolitis (NEC) is a severe inflammatory intestinal disease that occurs in nearly 7% of preterm neonates born at <1500 g annually (Nino et al., 2016). These premature infants have lower numbers of mature goblet cells and Paneth cells relative to term infants and, therefore, are more susceptible to infections by potentially pathogenic Gram-negative bacteria. In NEC patients, the pathogenic bacteria activate Toll-like receptor 4 signaling on the intestinal epithelium leading to the induction of an inflammatory cascade, which eventually results in reduced barrier integrity and patchy intestinal epithelial injury (Nino et al., 2016). The exact mechanisms underlying the destructive process in 10.13039/501100008010NEC are not fully understood but accumulating evidence supports the involvement of intestinal macrophages. Smad7 is up-regulated by macrophages infiltrating the mostly damaged intestine of NEC patients as well as in an experimental model of NEC induced in formula-fed mice by hypoxia and hypothermia (MohanKumar et al., 2016). In such a model, bacterial products stimulate Smad7 induction in neonatal macrophages, and Smad7, in turn, increases the sensitivity of macrophages to bacterial products, with the downstream effect of promoting NF-κB activation and cytokine production. Moreover, Smad7 induces IKK-β expression in macrophages through direct binding and transcriptional activation of the IKK-β promoter, and IKK-β increases further Smad7 level, thereby triggering a positive feedback loop, which amplifies the inflammatory activation of macrophages in NEC (MohanKumar et al., 2016). In line with this, studies in a model of NEC preterm baboons, which is caused by deficiency of TGF-β2 in the developing intestine, showed increased expression of Smad7. Notably, Smad7 can bind to TGF-β2 promoter and suppress TGF-β2 expression thus making a strong contribution to the development of NEC (Namachivayam et al., 2013).

Refractory celiac disease (RCD) is a severe form of coeliac disease, in which symptoms and signs of malabsorption, and villous atrophy can persist or recur despite a strict gluten-free diet (Hujoel and Murray, 2020; Marafini et al., 2020), probably as a result of excessive mucosal production of inflammatory cytokines (Caruso et al., 2014b). RCD is characterized by overexpression of Smad7 and reduced phosphorylation of Smad2/3 (Sedda et al., 2017), suggestive of defective TGF-β1 signaling. Knockdown of Smad7 with the AS in ex vivo organ mucosal explants of RCD patients leads to a marked downregulation of inflammatory cytokine production (Sedda et al., 2017).

Environmental enteropathy (EE) is an acquired inflammatory disease of the small intestine, characterized histologically by blunted villi, elongated crypts, and increased infiltration of the lamina propria with lymphocytes, and clinically associated with malabsorption and malnutrition signs (Syed et al., 2016). The fact that such pathological features resemble those seen in refractory CD prompted us to characterize the expression of Smad7 in children with EE. Analysis of Smad molecules in EE duodenal biopsies of children living in Pakistan and Zambia by Western blotting revealed higher levels of both Smad7 and phosphorylated Smad3 as compared to healthy controls. Immunohistochemical analysis showed that, in EE, Smad7 is expressed in both the epithelial and lamina propria compartments while phosphorylated Smad3 was expressed much more prominently in epithelial cells than in the lamina propria (Syed et al., 2018). Thus, the high Smad7 level and lack of phosphorylated Smad3 expression in the lamina propria suggest impaired TGF-β signaling in the lamina propria in EE similar to that previously reported in IBD and RCS.

The intestine is a major site of opportunistic infection by cytomegalovirus (CMV), which causes mucosal inflammation and in, some cases, severe organ dysfunction (Janoff and Smith, 2001; Kandiel and Lashner, 2006; Maher and Nassar, 2009). CMV is also the most common viral gastrointestinal pathogen in patients with IBD, even though it remains unclear whether the presence of CMV in the gut of IBD patients is indicative of a true infection or just a bystander. Indeed, it is known that, after primary infection, CMV typically persists in the bone marrow, infecting progenitor myeloid cells, which then enter the circulation as latently infected monocytes (Einhorn and Ost, 1984; Soderberg et al., 1993). These cells can eventually move to the intestine and differentiate into inflammatory cytokine-secreting macrophages (Genta et al., 1993; Sinzger et al., 1996; Smith et al., 1992; Wilcox et al., 1998). Infection of monocytes by CMV is associated with high induction of Smad7 that abrogates the TGF-β1-mediated NF-κB activation and NF-κB-dependent cytokine synthesis (Dennis et al., 2018). In line with this is the observation that Smad7 levels are up-regulated in patients with CMV-induced colitis and its expression declines after antiviral treatment (Dennis et al., 2018). Down-regulation of Smad7 in CMV-infected monocytes with AS restores TGF-β-induced immunosuppression (Dennis et al., 2018).

## 5 Clinical studies supporting the pathogenic role of Smad7 in Crohn’s disease

The findings discussed above paved the way for the development of an oral Smad7 AS-containing pharmaceutical compound, named initially GED0301, and later on mongersen (Monteleone et al., 2012). One phase 1 and 3 different phase 2 studies showed the clinical and endoscopic benefits of mongersen in patients with active CD (Monteleone et al., 2012, 2015; Feagan et al., 2018; Marafini et al., 2021). However, a phase 3, multicentre, double-blind, placebo-controlled study was discontinued after an interim analysis documented the lack of efficacy of the drug (Sands et al., 2020). The reasons for the discrepancy among these studies are not fully understood. The Smad7 rs144204026 C/T single nucleotide polymorphism (SNP) maps on the corresponding region targeted by the Smad7 AS contained in mongersen. Therefore, we examined whether such a variant allele could hamper the ability of Smad7 AS to bind and inhibit the expression of Smad7. No TT genotype was found in CD patients as well as in healthy volunteers. The heterozygous genotype was more frequent in CD patients as compared to controls but such a variant allele did not interfere with the inhibitory effect of the Smad7 AS, thus excluding the possibility that the presence of such a variant in CD patients enrolled in the phase 3 trial could have, at least in part, influenced the negative results (Di Fusco et al., 2020). A recent characterization of the chemical and pharmacological properties of the drug revealed that the stereochemistry was not homogenous among the batches developed during the whole clinical program and the batches used in phase 1 and 2 studies had stereochemistry that differed from that seen in most batches used in the phase 3 study. Moreover, most of the batches of mongersen used in the phase 3 study were not able to inhibit Smad7 expression in cultured cells (Arrico et al., 2022; Monteleone et al., 2022).

## 6 Conclusions

Accumulating evidence indicates that up-regulation of Smad7 expression is associated with defective TGF-β1-mediated immunosuppression in many inflammatory pathologies of the gut, thus suggesting a role for Smad7 in the amplification and perpetuation of detrimental signals in such disorders. Clinical studies with an oral Smad7 AS-containing pharmaceutical compound in patients with CD have provided us with conflicting results, and additional trials have been recently designed to further verify the efficacy of such an approach in clinical practice. Studies are also ongoing to establish which physical/chemical modifications, which could occur at the large-scale synthesis of the AS, have contributed to altering the pharmacological activity of some batches used in the phase 3 program, and how the manufacturing program can be improved to prevent additional issues in the future.

At the same time, further research work is warranted to clarify some points. For example, we do not yet know whether Smad7 is preferentially induced in some of the evolutive phases of IBD, and analysis of Smad7 in the inflamed gut of IBD patients helps identify better candidates for the treatment with mongersen. A recent study has shown that before initiation of therapy with TNF blockers and after 2 weeks of treatment, the expression of Smad7 in whole blood samples was decreased in IBD children who did not respond as compared to responders (Salvador-Martin et al., 2020), thus suggesting that Smad7 can be a potential marker for early response to anti-TNF in pediatric IBD patients. It remains also to be better evaluated whether in IBD there is a cell-specific regulation of Smad7 and which factors contribute to inducing Smad7 in the specific cell populations. In this context, for example, it remains to be ascertained whether the up-regulation of Smad7 in IBD epithelium can alter the epithelial barrier thus promoting a sequence of events that culminate in the tissue damage and its impact on the phases of mucosal healing. Finally, the fact that Smad7 expression is up-regulated in other intestinal inflammatory disorders could open additional possibilities for future therapeutic approaches.

## Funding

No specific funding has been received for this work.

## CRediT authorship contribution statement

**Giovanni Monteleone:** Writing – review & editing. **Federica Laudisi:** Writing – review & editing. **Carmine Stolfi:** Writing – review & editing.

## Declaration of competing interest

The authors declare the following financial interests/personal relationships which may be considered as potential competing interests: Giovanni Monteleone reports financial support was provided by First Wave BioPharma. Giovanni Monteleone reports a relationship with First Wave BioPharma that includes: consulting or advisory. Giovanni Monteleone has patent #treatment of inflammatory bowel diseases with Smad7 antisense oligonucleotides issued to Licensee. G Monteleone served as a consultant for First Wave BioPharma.

## Data Availability

No data was used for the research described in the article.
